# Evaluation of antitumor property of extracts and steroidal alkaloids from the cultivated Bulbus *Fritillariae ussuriensis* and preliminary investigation of its mechanism of action

**DOI:** 10.1186/s12906-015-0551-5

**Published:** 2015-02-21

**Authors:** Dongdong Wang, Yun Jiang, Ke Wu, Shu Wang, Yitao Wang

**Affiliations:** Department of Pharmacognosy, West China College of Pharmacy, Sichuan University, No. 17, Duan 3, Renmin Nan Road, Chengdu, 610041 Peoples’ Republic of China; State Key Laboratory of Quality Research in Chinese Medicine, Institute of Chinese Medical Science, University of Macau, Macau, 999078 Peoples’ Republic of China

**Keywords:** Bulbus *Fritillariae ussuriensis*, Alkaloids, Antitumor, Cell cycle, Microvessel density, Caspase-3

## Abstract

**Background:**

Cancer is well known as a leading cause of death in the world. At present, it is the very active area to search for anticancer drugs from natural products. In this study, we evaluated the antitumor property of chloroform extract (CE), n-hexane extract (HE), water extract (WE) and steroidal alkaloids from the cultivated Bulbus *Fritillariae ussuriensis* (BFU) and its preliminary mechanism for its action was investigated.

**Methods:**

Firstly, cytotoxicity of the different extracts from BFU against Lewis lung carcinoma cell line (LLC), Human ovarian cancer cell line (A2780), human hepatocellular carcinoma cell line (HepG2), human lung carcinoma cell line (A549) was measured by MTT assay. Then, we identified the compounds from the active extract of BFU by bioassay guided isolation, determined their antitumor activity *in vitro*, and detected cell cycle distribution using flow cytometry. Moreover, the extract of BFU which showed remarked anti-proliferative activity *in vitro* was further evaluated using S180 and LLC tumor models. Additionally, a preliminary investigation of the mechanism of the action was carried out by using histological and immunohistochemical staining technique.

**Results:**

The results showed that CE and the purified total alkaloids of BFU (TAFU) exhibited stronger cytotoxic activity than the others (WE and HE). We further isolated the four main steroidal alkaloids from TAFU, and found all alkaloids showed significant cytotoxicity, and peimisine induced G_0_/G_1_ phase arrest and increased apoptosis. The results showed that TAFU had significant antitumor activity and low toxicity *in vivo*. Additionally, the immunohistochemical examinations signified that TAFU remarkably increased caspase-3 expression and reduced microvessel density (MVD) in tumor tissues of transplantable S180 and LLC tumor models.

**Conclusions:**

These results achieved suggested that the steroidal alkaloids could hold a good potential for use as an antitumor drug. Notably, our finding is the first report on the antitumor activity of extracts and steroidal alkaloids from the cultivated BFU *in vitro* and *in vivo* and its mechanisms.

## Background

Bulbus *Fritillariae ussuriensis* (BFU) is the well-known food and folk medicine distributed in the Northeast Provinces of China, such as Liaoning, Heilongjiang and Jiling provinces, because it has remarkable antitussive, expectorant and antiasthmatic activities [1].

The previous studies have exhibited that the crude alkaloid extracts of BFU possess remarkable antitussive, expectorant and antiasthmatic activities [2,3]. *In vitro* previous studies suggested that verticinone, imperialine and peimisine from BFU inhibited angiotensin I converting enzyme activity in a dose-dependent manner [4], and *in vivo* ethylacetate and butanol extracts from BFU significantly lowered the mean arterial pressure, decreased angiotensin converting enzyme and angiotensin I-induced vasoconstriction, and they increased nitric oxide (NO) and cGMP productions in intact vascular tissue [5]. In addition, verticinone from BFU could induce Leukemia HL-60 cells to differentiate toward granulocytes *in vitro* [6]. Moreover, ethanol extract of BFU inhibited the production of inflammatory cytokine and MAPKs in mast cells [7]. In our previous study, imperialine, chuanbeinone, verticinone, verticine, imperialine-β-N-oxide, isoverticine and isoverticine-β-N-oxide isolated from genus *Fritillaria* have significant anti-inflammatory effect *in vivo* [8,9].

Cancer is well known as a leading cause of death in the world. It is predicted that cancer related deaths will increase to over 24 million in 2035 [10]. So, it is the very active area to search for anticancer drugs from natural products. It is worthwhile to note that several recent research have indicated that verticine, verticinone, ebeiedine and the crude alkaloid of *Fritillaria ebeiensis* showed strong antitumor activity in inhibiting the growth of the solid type of hepatoma and Ehrlich ascites carcinoma in mice [11]. Besides, the crude alkaloid extract of bulbs of *Fritillaria puqiensis* and puqietinone could inhibit the growth of three types of cancers [12]. Moreover, our previous studies also suggested that the crude alkaloid extract of bulbs of *Fritillaria Cirrhosae* showed remarkable antitumor *in vitro* and *in vivo* [13,14].

To the best of our knowledge, there are few publications focusing on the antitumor activity of different extracts of BFU *in vitro* and *in vivo*. Therefore, in this study, we prepared different extracts (chloroform extract (CE), n-hexane extract (HE), water extract (WE) and total alkaloids of BFU (TAFU)) from BFU and assessed their antitumor activity *in vitro*. Furthermore, we identified the compounds from the active extract of BFU by bioassay guided isolation, and determined their antitumor property *in vitro*. Moreover, the extract of BFU which showed remarked anti-proliferative activity *in vitro* was further evaluated *in vivo*. Additionally, preliminary mechanism of its action was investigated.

## Methods

### Chemicals and reagents

RPMI-1640 medium, Dulbecco’s Modified Eagle’s medium (DMEM) and fetal bovine serum (FBS) were purchased from Hyclone (USA). Trypsin, penicillin, streptomycin, antibodies, propidium iodine (PI) and phosphate buffer solution (PBS) were purchased from Gibco BRL, Life Technologies (USA). 3-(4,5-Dimethylthiazol-2-yl)-diphenyl tetrazolium bromide (MTT), dimethyl sulphoxide (DMSO) were purchased from Sigma Aldrich, Inc. (USA). Mitozantrone hydrochloride was purchased from Jiangsu Hansoh Pharmaceutical Co., Ltd (China). Cyclophosphamide (CTX) was purchased from Jiangsu Hengrui Co. Ltd (China). The ultra pure water was prepared by a Milli-Q water system (China). Sterilized cell culture materials, such as syringe filter, 15 ml and 50 ml tubes, 96-well plates, and pipettes were purchased from Nest Biotech Co., Ltd (China). Other chemicals and reagents used were analytical grade and commercially available.

### Plant materials

The BFU were purchased in September 2012 from Chengdu International Trade City Hehuachi Chinese Medicinal Herbal Market (Chengdu city, Sichuan province, China) and identified by Prof. Shu Wang (Department of Pharmacogonosy, West China College of Pharmacy, Sichuan University). The sample (W201204001) has been deposited in the pharmacognosy laboratory of West China College of Pharmacy, Sichuan University.

### Extraction and isolation

The different extracts of BFU were prepared according to previously described methods [14]. Briefly, the dried powder was immersed in ammonia, and then extracted with EtOH solvent by maceration for 6 hours. After filtration, the solvent was evaporated to obtain EtOH extract (EE). The EE was dissolved in 3% HCl and then partitioned with petroleum ether. The pH of the aqueous solution was readjusted to 10.0 and extracted with chloroform and n-hexane in sequence. Each of these fractions was evaporated to yield petroleum ether extract (PE), chloroform extract (CE), n-hexane extract (HE) and water extract (WE).

From the previous research, we could conclude that the CE had more total alkaloids [13,14]. The CE which mainly contained total alkaloids showed the highest antitumor activity *in vitro* among three extracts (CE, HE and WE). So, the CE was subjected to further purification to get total alkaloids of BFU (TAFU). TAFU was prepared by using previous methods [15]. Simply, the CE was dissolved and chromatographed over H-103 resin column, which was eluted with distilled water, 10% alcohol and 90% ethanol, respectively. The fraction eluted with 90% alcohol was collected and lyophilized, and the resulting powder (TAFU) was subjected to the determination of total alkaloids contents, isolation and pharmacological studies.

The TAFU was separated repeatedly by silica gel column chromatography using petroleum ether-acetone-diethylamine (6:1:1 ~ 1:1:1) of increasing polarity as eluent as reported [8,9]. 15-ml fractions were collected throughout and combined on the basis of Thin Layer Chromatography (TLC). The alkaloids isolated from TAFU were determined by comparing the samples’ melting point and spectral data (^1^H-, ^13^C-NMR spectra) with those reported in literature [9,11,16].

### Measurement of total alkaloids content

The total alkaloids content was determined according to previously described method [13]. For alkaloids standard, a stock solution of imperialine was prepared and diluted to give working standards. For samples, the adequate CE and TAFU were dissolved in chloroform. An aliquot of each sample or standard solution was stained with bromocresol green buffer solution to measure total alkaloids content. Absorbance was measured at 412 nm using Alpha-1900PC UV–Vis spectrophotometer (Shanghai Lab-Spectrum Instruments Co., Ltd., China). Data were reported as mean ± standard error of mean (S.E.M.).

### Cell lines and cell culture

Lewis lung carcinoma cell line (LLC), Human ovarian cancer cell line (A2780), human hepatocellular carcinoma cell line (HepG2), and human lung carcinoma cell line (A549) were obtained from Key Laboratory of Drug Targeting and Drug Delivery System, Ministry of Education, Sichuan University (Chengdu, China). These cell lines were grown and maintained in a humidified incubator at 37°C and in 5% CO_2_ atmosphere. RPMI-1640 and DMEM (high glucose) medium were supplemented with 10% FBS, 100 units/ml penicillin and 100 μg/ml streptomycin. A549 cells were maintained with RPMI1640, A2780, HepG2 and LLC cells were cultured with DMEM.

### Cytotoxicity assay

The cytotoxicity of the different kinds of extracts against the tumor cells was assessed via MTT assay [13,17-20]. HepG2 cells were seeded into six wells at a density of 6×103 cells per well in 96-well microplates, and A2780, A549 and LLC cells were seeded at a density of 8×103 cells per well. Cells were permitted to adhere for 24 h, and then treated with the various concentrations of CE, HE, WE or TAFU for 72 h. DMSO was used to dissolve the extracts, and its final concentration was maintained at 0.5% (v/v). The cultured medium was removed and replaced with 150 μl MTT (0.5 mg/ml) per well before termination at 4 h. After removal of the MTT solution, 200μl DMSO was added to each well. The absorbance was recorded on a Thermo Scientific microplate spectrophotometer (Thermo Fisher Scientific Inc., USA) at the wavelength of 490 nm. The concentration of the extracts which gives 50% growth inhibition is referred to as the IC50 (μg/ml), which was calculated for each extracts from the dose-response curves [13,14].

Moreover, the serial concentrations of TAFU were used to treat the tumor cells for 24, 48 and 72 h to test the time-dependent cytotoxicity. Furthermore, we measured IC50 of four alkaloids monomers (imperialine, peimisine, verticine and verticinone) for these tumor cells by MTT assay as mentioned above. All experiments were performed independently in triplicate and data were presented as mean ± S.E.M.

Changes in cell morphology were checked visually under phase contrast microscope (Carl Zeiss Axiovert 40 CFL, Germany) after incubation with IC_50_ concentrations of TAFU for 24, 72 h, where untreated cells served as control.

### Quantitative detection of apoptosis and cell cycle distribution

The cell cycle was analyzed using flow cytometry as previously described [14,21]. A2780 cells (5.0 × 10^5^ cells/well) were seeded in 6-well plate and grown until they reached 80% confluence. Then, the cells were treated with peimisine (15 μg/ml) for 24, 48 and 72 h. The cells were harvested, washed thrice with cold PBS and fixed with 1.0 ml of 70% ethanol at 4°C overnight. Then, cells were washed with PBS and incubated in 1.0 ml of PBS containing 100 μg PI, 100 μg RNase A in darkness for 30 min at 37°C, and sorted in a FACScan flow cytometry using Kaluza 1.1 software (Bechman Coulter Inc., USA). Cell cycle distribution and sub-G_1_ groups were calculated using ModFit LT software. We did not evaluate verticinone for cell cycle distribution because we did not have sufficient amount of verticinone to perform this experiment.

### Animals and establishment of tumor model

Male ICR mice and C57BL/6 J mice weighing 18–22 g were purchased from Experimental Animal Center of West China College of Pharmacy, Sichuan University (Certificate No. SCXK (Chuan) 2012–09, Chengdu, China). All mice were housed in specific pathogen-free (SPF) level laboratory with a 12 h light–dark cycle, and provided sterile food and water *ad libitum* [22]. All the procedures were in strict accordance with the Chinese legislation on the use and care of laboratory animals and the guidelines established by Institute for Experimental Animals of Sichuan University and were approved by the Sichuan University Committee on Animal Care and Use.

Sarcoma 180 (S180) tumor model was established following the protocols previously described [9,13,23,24]. S180 cells were maintained in the ascitic form by sequential passages in ICR mice. A mouse bearing a 7-day S180 was sacrificed. The cells were pipetted out and diluted with PBS to reach the final concentration of 1 × 10^7^ cells/ml. A volume of 0.2 ml S180 cells suspension was subcutaneously inoculated into the left axilla of ICR mice to establish the S180 tumor model.

LLC tumor model was established based on the methods previously described [14,23,25]. LLC cells suspension was subcutaneously inoculated into the left axilla of C57BL/6 J mice (2 × 10^6^ cells/mouse). After 7–10 days, the solid tumor was removed from the mice and single cells were prepared by mincing tumor fragments. The cells were adjusted to 1 × 10^7^ cells/ml and a volume of 0.2 ml LLC cells suspension was subcutaneously inoculated into the left axilla of C57BL/6 J mice to establish the LLC tumor model.

### Measurement of xenograft tumour growth inhibition

The tumor model animals were randomly divided into five groups of 10 mice each at 24 h after tumor inoculation, group 1 (Control, 1% Tween 80 solution, 0.2 ml/20 g/day), group 2 (the positive control, CTX, 20 mg/kg/day), group 3 (the low dose of the TAFU, 20 mg/kg/day), group 4 (the medium dose of the TAFU, 40 mg/kg/day), group 5 (the high dose of the TAFU, 80 mg/kg/day). The dose selection of TAFU is based on the previous experiments. The mice were orally administered drugs once a day for 10 days. On the 11th day, the mice were weighed, sacrificed, and then tumor, thymus, spleen, heart, liver, lung and kidney were collected to detect inhibition rate of tumor and organ indices. Then, the tumor tissues were fixed in 4% neutral buffered paraformaldehyde immediately for the histological and immunohistochemistry examination. The *in vivo* antitumor activity of TAFU was expressed as an inhibition ratio: Inhibition (%) = (C_w_ − T_w_)/C_w_ × 100, where C_w_ is the average tumor weight of the control group, and T_w_ is the average tumor weight of tested group. The organ index was calculated as follows: Organ index (mg/g) = organ weight/(body weight − tumor weight) [13,26,27].

### Histological and immunohistochemical examinations

The histological evaluation was performed as described previously [14,28]. All tumor tissues were randomly cut into 10 histological sections and stained with hematoxylin and eosin (H&E). Histological examinations were carried out by light microscopy (Nikon Eclipse E100).

Immunohistochemical detection of caspase-3 and CD31 was carried out by standard immunohistochemical techniques [14,29]. Briefly, these sections were incubated with 1% H_2_O_2_ to inactivate endogenous peroxidase and put into 10 mM sodium citrate buffer solution to recover antigen. Non-specific binding sites were blocked for 1 h in PBS containing 1.5% normal serum. Then, the slides were incubated with primary caspase-3 and CD31 antibody at 4°C overnight, and then incubated with biotinylated secondary antibody for 20 min, followed by incubation with streptavidin-horseradish peroxidase for 20 min. The immune reactions were visualized by immersing the slides in 3,3’-diaminobenzidine tetra hydrochloride reagent. The slides were counterstained with hematoxylin and then dehydrated with sequential ethanol for sealing.

We performed immunohistochemical detection of CD31 in tumor sections to determine the antiangiogenesis effects. Tumor microvessel density (MVD) was quantified for high dose group, CTX group and Control group. MVD was determined by counting the number of microvessels per high-power field in the sections as previously described [14,30].

Immunohistochemistry assessment of caspase-3 expression was conducted to investigate the apoptosis of tumor cells. Immunohistochemically positive cells for casaspe-3 showed brown granules. Integrated optical density (IOD) of brown color of every field of view was quantified using Image-Pro plus software (Media Cybernetics Inc., USA) as previously described [14,31].

### Statistical analysis

The results were expressed as means ± S.E.M.. Statistical analysis was performed by a one-way analysis of variance ANOVA test for multiple group comparison and by Student’s *t*-test for comparison of two groups using the SPSS statistics 17.0 software package (LEAD Technologies, Inc., USA). A value of *p* < 0.05 was considered to be statistically significant.

## Results

### Extraction, isolation and content of total alkaloids of the extracts

**Figure 1:**
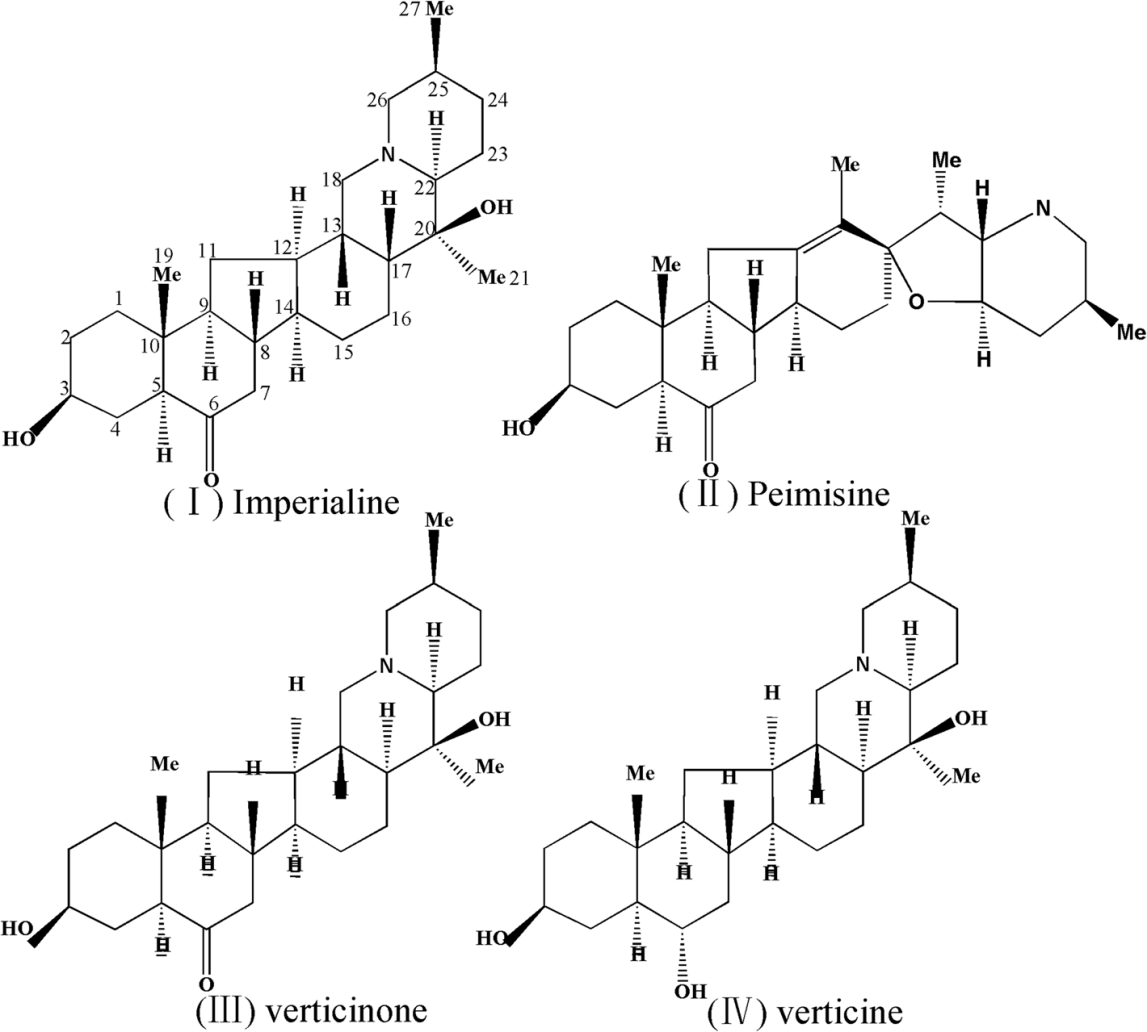
**The molecular structures of four steroidal alkaloids from BFU.** The steroidal alkaloids are imperialine (I), peimisine (II), verticinone (III), verticine (IV).

BFU was extracted and fractioned to afford EE (15.27%), PE (0.021%), CE (1.028%), HE (7.692%) and WE (0.660%). Four steroidal alkaloids were obtained from TAFU and identified as imperialine (I), peimisine (II), verticinone (III), and verticine (IV) (Figure 1) based on their physical and spectral data which showed good agreement with published references [9,11,12]. The structures of the four compounds presented in Figure 1. In addition, the total alkaloids contents in CE and TAFU were determined as 19.67% and 54.37%, respectively.

### Evaluation of cytotoxicity against tumor cells

**Figure 2:**
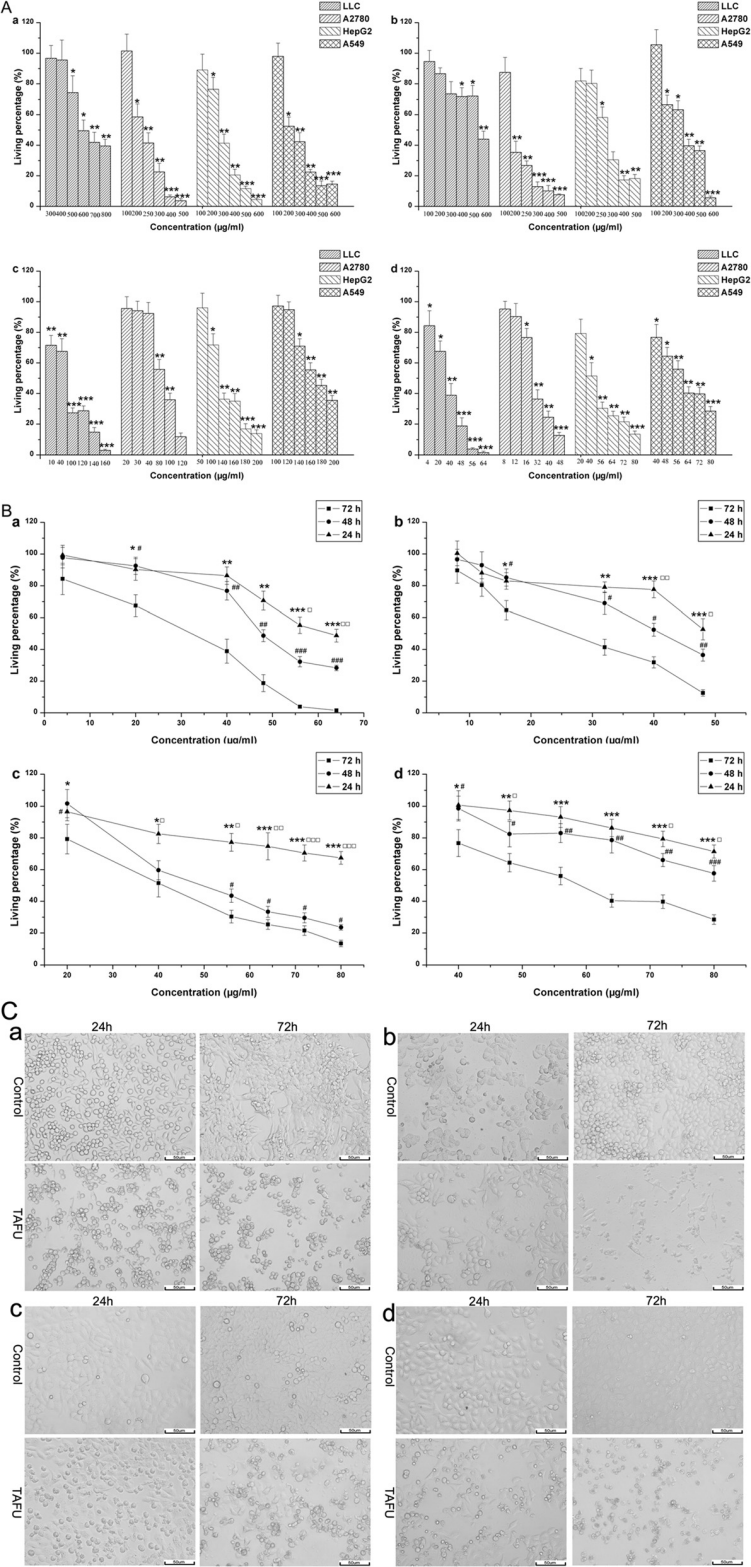
**The cytotoxic effects of the different fractions against tumor cells. (A)**, Effects of different concentrations of HE, WE, CE and TAFU on proliferation of LLC, A2780, HepG2 and A549 cells. **(a)**, HE; **(b)**, WE; **(c)**, CE; **(d)**, TAFU. Three cell lines were treated with four fractions for 72 hours. Significant differences compared with Control group were designated as ^*^P < 0.05, ^**^P < 0.01 and ^***^P < 0.001. **(B)**, Time and dose effects of different concentrations of TAFU on LLC, A2780, HepG2 and A549 cells growth. **(a)**, LLC; **(b)**, A2780; **(c)**, HepG2; **(d)**, A549. Significant differences of inhibitory effects between treatment of 24 h and 72 h were designated as ^*^P < 0.05, ^**^ P < 0.01 and ^***^P < 0.001; Significant differences of inhibitory effects between treatment of 24 h and 48 h were designed as ^□^P < 0.05 ^□□^P < 0.01 and ^□□□^P < 0.001; Significant differences of inhibitory effects between treatment of 48 h and 72 h were designed as ^#^P < 0.05, ^##^P < 0.01 and ^###^P < 0.001. **(C)**, Microscopic image of TAFU (IC_50_) treated LLC, A2780, HepG2 and A549 cells for 24 and 72 hours. Cells morphology was observed by a phase contrast inverted microscope (200×). (a), LLC; (b), A2780; **(c)**, HepG2; **(d)**, A549. All results were expressed as the mean ± S. E. M. (n = 3).

To evaluate cytotoxicity of the different fractions against tumor cell lines, the MTT assay was used. The cytotoxic effects of HE, WE, CE and TAFU on LLC, A2780, HepG2 and A549 cells were examined at different concentrations (Figure 2A), and their IC_50_ were determined (Table 1). The results suggested that CE and TAFU showed the significantly higher cytotoxic effects than HE and WE, especially TAFU showed the most effective inhibitory activity. In addition, two cancer cell lines (A2780 and LLC) were more sensitive to the CE and TAFU than the others. Moreover, we evaluated the cytotoxicity of verticine, vericinone, imperialine and peimisine *in vitro*, and their IC_50_ are shown in Table 1. The four alkaloids showed significant cytotoxic effects, and verticinone and peimisine showed markedly higher inhibitory effects than the others. To our knowledge, different fractions of BFU and the alkaloids were firstly studied regarding the cytotoxic activity against these tumor cell lines.

**Table 1:** **IC**_**50**_ **values of the different fractions and alkaloids from BFU against tumor cell lines**

**Fractions/compounds**	**IC**_**50**_ **(μg/ml)**			
	**LLC**	**A2780**	**HepG2**	**A549**
HE	660.11 ± 16.71	246.97 ± 18.78	288.95 ± 17.84	294.96 ± 24.46
WE	614.74 ± 19.55	208.38 ± 23.57	279.09 ± 13.06	461.26 ± 28.38
CE	97.55 ± 4.02	88.50 ± 5.26	128.26 ± 12.55	172.59 ± 6.59
TAFU	39.37 ± 2.36	27.65 ± 1.84	51.10 ± 2.84	58.60 ± 3.99
Verticine	16.53 ± 0.82	48.41 ± 2.58	53.38 ± 3.16	97.58 ± 3.72
Vericinone	3.03 ± 0.32	17.03 ± 0.89	14.64 ± 0.65	47.39 ± 1.90
Imperialine	66.26 ± 3.46	39.18 ± 2.21	84.26 ± 4.46	117.84 ± 6.29
Peimisine	20.75 ± 1.44	17.43 ± 0.80	92.07 ± 4.89	36.11 ± 1.70

The reported values are the means ± S.E.M. (n = 3).

The cytotoxic activities of TAFU against the four tumor cell lines at series concentrations with different treatment time were studied. As shown in Figure 2B, TAFU had cytotoxicity against the four kinds of tumor cells in a time-dependent manner.

After incubation with IC_50_ concentrations of TAFU for 24, 72 h, its effects on cell morphology are shown in Figure 2C. Compared with the negative control, classical apoptotic cells were observed in TAFU mediated cell death.

### Effect of TAFU on tumor growth and organ indices in tumor-bearing mice

To further evaluate the antitumor effects of TAFU, an *in vivo* antitumor study using S180 and LLC tumor models were performed. The inhibitory effects of TAFU at the high (80 mg/kg/day), medium (40 mg/kg/day) and low dose (20 mg/kg/day) on the transplanted S180 and LLC tumors are shown in Table 2. The higher dose of TAFU significantly inhibited the growth of transplantable S180 sarcoma and LLC tumors in mice in dose-dependent manner. As a positive control drug, CTX showed higher inhibitory rate.

**Table 2:** **Inhibitory effects of the TAFU on the tumor growth of S180 and LLC tumor models**

**Groups**	**Dose (mg/kg)**	**No. of animals**	**Weight of tumor (g)**	**Inhibition (%)**
S180 tumor model				
Control	-	10	1.20 ± 0.30	-
TAFU	20	10	1.22 ± 0.21	−1.46
	40	10	0.99 ± 0.10^*^	17.80
	80	10	0.62 ± 0.06^**^	48.56
CTX	20	10	0.53 ± 0.13^***^	55.92
LLC tumor model				
Control	-	10	2.27 ± 0.16	-
TAFU	20	10	2.38 ± 0.17	−4.68
	40	10	2.11 ± 0.24	7.22
	80	10	1.36 ± 0.20^**^	39.96
CTX	20	10	0.94 ± 0.11^***^	58.37

The reported values are the means ± S.E.M. (n = 10). Significant differences compared to Control group are indicated by ^*^P < 0.05, ^**^P < 0.01 and ^***^P < 0.001.

As shown in Table 3, body weight of animals which received TAFU has no significant difference compared with that of Control group. In contrast, the body weight of mice with treatment of CTX significantly decreased compared to that of TAFU- and non-treatment. In addition, immune organs (the thymus and spleen) indices in the CTX-treated group were significantly lower than that of the TAFU and Control groups. The result indicated that TAF had significant antitumor activity *in vivo*, and lower toxicity compared with CTX on the basis of body weight and organ indices.

**Table 3:** **Organ weights index of the mice xenografted with S180 cells and LLC cells**

**Groups**	**Dose (mg/kg)**	**Growth of weight (g)**	**Organ indices (mg/g)**					
			**Spleen**	**Thymus**	**Heart**	**Liver**	**Lung**	**Kidney**
S180 tumor model								
Control	-	4.08 ± 0.61	7.02 ± 0.70	2.24 ± 0.22	5.17 ± 0.40	63.71 ± 3.53	6.88 ± 0.52	13.01 ± 0.61
TAFU	20	2.36 ± 0.81^###^	6.86 ± 0.20^##^	2.43 ± 0.35^##^	4.34 ± 0.12	63.28 ± 2.10^#^	7.29 ± 0.37	13.00 ± 0.50
	40	2.84 ± 0.74^#^	6.67 ± 0.17^##^	1.98 ± 0.12^*, #^	4.67 ± 0.20	56.04 ± 1.11^*^	6.62 ± 0.21	12.29 ± 0.37
	80	2.45 ± 0.50^##^	5.43 ± 0.11^*, #^	1.84 ± 0.17^*^	4.55 ± 0.30	58.50 ± 0.82	6.39 ± 0.45	12.88 ± 0.63
CTX	20	−1.82 ± 0.64^***^	4.26 ± 0.16^***^	1.30 ± 0.15^**^	4.73 ± 0.18	52.72 ± 3.30^*^	6.75 ± 0.39	12.81 ± 0.59
LLC tumor model								
Control	-	0.64 ± 0.48	6.07 ± 0.91	1.57 ± 0.32	5.93 ± 0.48	57.54 ± 3.99	6.90 ± 0.52	13.63 ± 0.60
TAFU	20	0.58 ± 0.68^###^	7.74 ± 0.57^##^	1.25 ± 0.13^###^	5.22 ± 0.32^#^	58.69 ± 1.32^#^	7.20 ± 0.33^*^	12.54 ± 0.55
	40	0.37 ± 0.32^*, ##^	7.53 ± 0.38^##^	1.18 ± 0.04^##^	5.25 ± 0.43^#^	53.44 ± 1.20^#^	7.33 ± 0.10	11.17 ± 0.22
	80	0.29 ± 0.50^*, ##^	5.68 ± 0.82^#^	1.09 ± 0.07^*, ##^	4.86 ± 0.12^*^	53.85 ± 4.96^#^	6.87 ± 0.60^#^	11.69 ± 0.40
CTX	20	−1.88 ± 0.71^***^	4.05 ± 0.42^**^	0.41 ± 0.05^***^	4.38 ± 0.17^*^	44.34 ± 3.59^*^	6.80 ± 0.54	11.96 ± 0.62

The reported values are the means ± S.E.M. (n = 10). Significant differences compared with Control group were designated as ^*^P < 0.05, ^**^P < 0.01 and ^***^P < 0.001; and those compared with CTX group as ^#^P < 0.05, ^##^P < 0.01 and ^###^P < 0.001.

### Peimisine induced G_0_/G_1_ phase arrest and rising apoptosis rate

**Figure 3:**
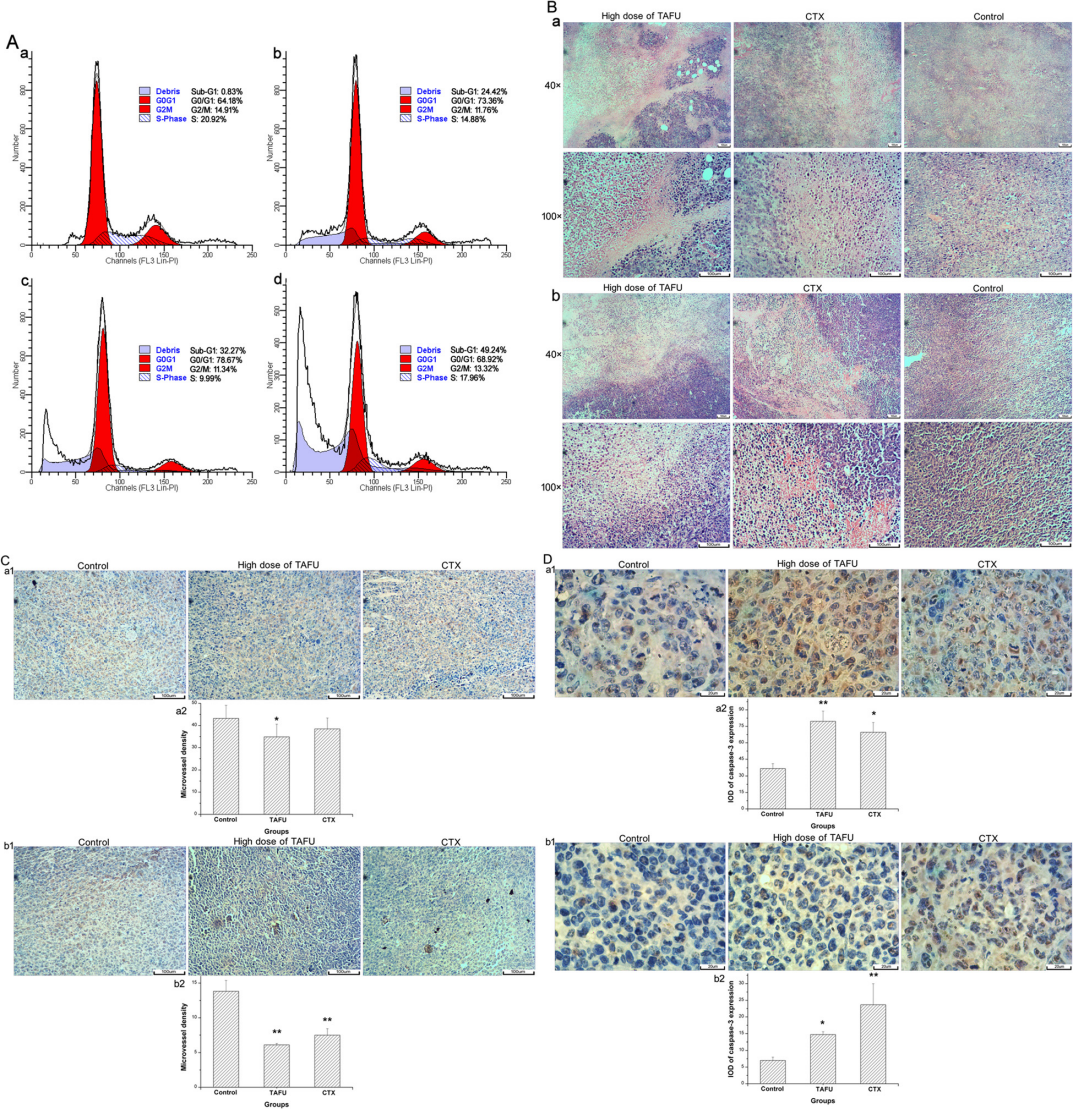
**Mechanism of antitumor activity of alkaloids from BFU. (A)**, Effects of peimisine on A2780 cells cycle distribution and the apoptosis rate. The cells were treated with peimisine (15 μg/ml) for 0, 24, 48 and 72 h. **(a)**, 0 h; **(b)**, 24 h; **(c)**, 48 h; **(d)**, 72 h. **(B)**, Histopathological examination of tumor tissue in S180 and LLC tumor models treated with TAFU, CTX and Tween 80 solution as Control by HE staining images (magnification: 40× and 100×), and there are larger areas of necrotic region in the high dose of TAFU and CTX groups than that of Control group. **(a)**, tumor tissue in S180 tumor model; (b), tumor tissue in LLC tumor model. **(C)**, Effects of TAFU on CD31 expression in mice treated with TAFU, CTX and Tween 80 solution as Control. Typical images of immunohistochemical staining of CD31 in tumor mice (magnification: 100×), and the positive cells were stained by brown. **(a1)**, tumor tissue in S180 tumor model; **(a2)** microvessel density (MVD) of tumor tissue in S180 tumor model; **(b1)**, tumor tissue in LLC tumor model; **(b2)** MVD of tumor tissue in LLC tumor model. **(D)** Effects of TAFU on caspase-3 expression in mice treated with TAFU, CTX and Tween 80 solution as Control. Typical images of immunohistochemical staining of caspase-3 in tumor mice (magnification: 400×), and the positive cells were stained by brown. (a1), tumor tissue in S180 tumor model; **(a2)** IOD of caspase-3 expression in tumor tissue of S180 tumor model; **(b1)**, tumor tissue in LLC tumor model; **(b2)** IOD of caspase-3 expression in tumor tissue of LLC tumor model. All results were expressed as the mean ± S. E. M., significant differences compared with Control group were designated as ^*^P < 0.05 and ^**^P < 0.01.

To investigate the effect of peimisine on the apoptosis and the cell cycle distribution of A2780 cells, flow cytometry was used. As depicted in Figure 3A, the typical sub-G_1_ peak, which represented the apoptosis cell population, appeared in the DNA content after the treatment with peimisine. The percentage of apoptosis increased in a time-dependent manner and the changes in the cell cycle progression were notable (Figure 3A). Besides, the cell population of the S phase reduced concurrently with the increase of the G_0_/G_1_ phase population after the treatment with peimisine. The results suggested that peimisine mainly induced G_0_/G_1_ phase arrest of A2780 cells in a time-dependent manner, which might be contributing to the apoptosis.

### Effects of TAFU on histological changes, CD31 and caspase-3 expression of tumor tissues

In order to further investigate the mechanisms of antitumor efficacy of TAFU, HE CD31 and caspase-3 immunohistochemical staining were performed on tumor tissues. The morphology of apoptosis cell in tumor tissues was observed by HE staining technique. The results suggested that apoptotic cells observed in the TAFU- or CTX-group were more significant in tumor tissues compared with that in the control group (Figure 3B). In Control group, the tumor architecture was intact and clear, and the tumor cells showed obvious nucleolus cleavage and high extent of malignancy.

We investigated the anti-angiogenic effects of TAFU by immunostaining of tumor sections for the endothelial cell marker CD31, which is the most specific and sensitive endothelial marker. Figure 3C shows the typical reduction of tumor blood vasculature number after TAFU and CTX treatment. As show in Figure 3C, the positive cells were counted from five different areas for each sample, and there were 34.85 ± 5.74, 38.45 ± 4.90 and 43.10 ± 5.89 for the higher dose of TAFU, CTX and Control groups in S180 tumor model respectively, and 6.10 ± 0.21, 7.50 ± 0.93 and 13.85 ± 1.57 for the higher dose of TAFU, CTX and Control groups in LLC tumor model, respectively.

Immunohistochemical analysis of caspase-3 expression in solid tumor is shown in Figure 3D. A profound increase in caspase-3 expression was observed in solid tumors from mice treated with TAFU and CTX. As show in Figure 3D, integrated optical density (IOD) of brown color of every observed field was quantified from eight representative fields for each sample, and there were 79.57 ± 9.37, 69.60 ± 8.88 and 36.77 ± 4.23 for the higher dose of TAFU, CTX and Control groups in S180 tumor model respectively, and 14.71 ± 0.83, 23.65 ± 6.33 and 7.01 ± 0.97 for the higher dose of TAFU, CTX and Control groups in S180 tumor model, respectively.

## Discussion

In recent years, increasing attention has been paid to genus of *Fritillaria* owing to its powerful potential therapeutic benefits for various diseases. Previous studies have reported that the total alkaloids or monomers of alkaloids from the genus of *Fritillaria* (*Fritillaria ebeiensis* and *Fritillaria cirrhosae*) have antitumor activities [11,13,14]. It is well known that some plants of the genus *Fritillaria* are on the edge of extinction which is regarded as major constraint for further development and utilization [32]. However, there are very mature artificial plantation technologies for BFU, the cultivated BFU become the mainstream of BFU in market [33], which provides the adequate resources for further exploitation and utilization of BFU. Moreover, antitumor effects of BFU have not yet been properly investigated. So, it was our primary goal to evaluate antitumor activity of BFU *in vitro* and *in vivo* and explore its related mechanism for its action.

Cytotoxic assay is used to search which fractions have potential antitumor properties. In our work, samples with higher total alkaloids (CE and TAFU) trended to have stronger cytotoxic activity against tumor cancer cells than the other two fractions (HE and WE), and they all inhibited cell proliferation in a dose- and time-dependent manner. The results suggested that the antitumor potential of the fractions might mainly be attributed to the total alkaloids content. Moreover, we isolated four main alkaloids (imperialine, peimisine, verticine and verticinone) from TAFU, measured their cytotoxic effects, and found that they all showed significant cytotoxic activity *in vitro*, which proved that the steroidal alkaloids are truly involved in the antitumor activity. Besides, our previous study showed that the alkaloids from BFU had no significant cytotoxic effect on normal ARPE-19 cells compared to DMSO at concentrations ranging from 10 to 320 μg/ml, and they showed that IC_50_ values of these compounds is higher than 200 μg/ml [34].

Apoptosis is an important biological mechanism that contributes to the maintenance of the integrity of multi-cellular organisms that is dependent on the expression of cell-intrinsic suicide machinery [14]. Apoptosis is characterised by key morphological features, such as cell shrinkage, membrane blebbing, chromatin condensation and generation of apoptotic bodies [35]. The results of this study showed typical morphological characteristics of apoptosis, such as nuclear condensation and apoptotic body formation, in tumor cells treated with TAFU. Furthermore, the apoptosis of A2780 cells induced by peimisine was also confirmed by the sub-G1 DNA accumulation. In addition, the results showed that peimisine induced an accumulation of cells in G_0_/G_1_ phase with an increasing apoptotic rate, which indicated that alkaloids from BFU induced apoptosis likely through G_0_/G_1_ phase arrest.

The further results of experiment *in vivo* showed that TAFU could significantly inhibit growth of transplantable S180 sarcoma and LLC tumor in mice. It is well known that most of chemical antitumor drug could causes toxic side effects, while natural products often have very low clinical toxicity compared with them [36]. In this study, the toxicity of TAFU was evaluated based on changes of body weight and organ indices in ICR and C57BL/6 J mice. These results showed that CTX markedly decreased body weight, thymus and spleen indices in mice as compared with TAFU group and Control group. As the important immune organs, the spleen and thymus indices reflect the immune function of the organism [14]. By combining the tumor weight, body weight and organ indices measurement, it was indicated that TAFU had significant antitumor activities *in vivo* and fewer side effects compared with CTX.

As indicated in many other studies, apoptosis is a type of cell death process regulated in an orderly way by a series of signal cascades under certain situations [13]. Caspases family plays a crucial role in the mechanism of cell apoptosis. To further clarify the mechanism of its antitumor activity, we investigated whether caspase-3, the major downstream effector of apoptosis [37], was involved in TAFU-induced apoptosis in tumor tissues. The results showed that TAFU significantly inhibited transplanted S180 and LLC tumors in a manner that causes caspase-dependent apoptosis by activating the caspase-3.

Relevant studies have demonstrated that tumor growth and metastasis are dependent on angiogenesis. So, inhibition of tumor angiogenesis may affect the tumor growth and decrease metastatic potential of tumors [38]. MVD has been considered as a useful prognostic indicator reflecting tumor angiogenesis. The intratumoral MVD was examined by immunohistochemical analysis to assess whether TAFU induced inhibition of xenograft tumor growth was mediated through its anti-angiogenic effects. Microscopic examination of tumors showed IOD of CD31 stained cells in TAFU- and CTX-treated group were markedly less than that of control group. The results suggested that the inhibit effects of TAFU on the number of microvessel may partially contribute to the inhibition of tumor growth in mice.

## Conclusions

In conclusion, this study showed investigation of cytotoxicity of different fractions from BFU *in vitro*, identification of the active alkaloids by bioassay guided isolation, determination of cytotoxic activity of steroidal alkaloids *in vitro*, measurement of the inhibitory effects of TAFU on xenograft tumor and its relative mechanism. CE and TAFU with higher total alkaloids exhibited notable cytotoxicity against the four tumor cell lines, and TAFU significantly inhibited the growth of transplantable S180 sarcoma and LLC tumor in mice with lower toxicity. In addition, four main steroidal alkaloids were isolated from TAFU and identified as imperialine, peimisine, verticinone and verticine. Furthermore, all alkaloids showed significant cytotoxicity against four tumor cells and peimisine induced G_0_/G_1_ phase arrest and increased apoptosis rate. Moreover, the histological examination indicated that TAFU could change the morphological features of tumor cells, induce their apoptosis. The immunohistochemical examinations showed that TAFU remarkably increased caspase-3 expression and reduced MVD in tumor tissues of transplantable S180 and LLC tumor models. Notably, our finding is the first report on the antitumor activity of extracts and steroidal alkaloids from the cultivated BFU *in vitro* and *in vivo* and its mechanisms, however, further studies are needed to elucidate the precise mechanisms of its antitumor activity.

## Abbreviations

BFP: Bulbus *Fritillariae ussuriensis*
CE: Chloroform extracts
CTX: Cyclophosphamide
DMEM: Dulbecco’s Modified Eagle’s medium
DMSO: Dimethyl sulphoxide
FBS: Fetal bovine serum
HE: n-hexane extracts
H & E: Hematoxylin and eosin
IC_50_: 50% inhibitory concentration
IOD: Integrated optical density
EE: EtOH extracts
MTT: 1-(4,5-Dimethylthiazol-2-yl)-3,5-diphenyl-formazan
MVD: Microvessel density
PI: Propidium iodine
TAFU: Total alkaloids of BFU
WE: Water extracts
